# The incremental yield of CMA over karyotype in fetal ventriculomegaly: a systematic review and meta-analysis

**DOI:** 10.1007/s00404-026-08470-8

**Published:** 2026-06-02

**Authors:** Ioakeim Sapantzoglou, Zacharias Fasoulakis, Konstantinos Tasias, Angeliki Rouvali, Afroditi Pegkou, Marianna Theodora, Georgios Daskalakis, Panagiotis Antsaklis

**Affiliations:** 1St Department of Obstetrics and Gynecology, Alexandra Hospital, National and Kapodistrian University of Athens, Vasilissis Sofias 80 Aven. 2-4, Lourou Str, 11528 Athens-Greece, PC Greece

**Keywords:** Fetal ventriculomegaly, VM, CMA, Array CGH, Karyotype

## Abstract

**Objective:**

The main objective of our study was to conduct a systematic literature review and a meta-analysis to evaluate the incremental yield of chromosomal microarray analysis compared to karyotyping in cases of fetal ventriculomegaly.

**Methods:**

Our review was designed according to the PRISMA guidelines. It included all observational studies that reported the results of CMA testing in fetuses diagnosed with ventriculomegaly (both isolated and non-isolated),in fetuses with isolated ventriculomegaly, in fetuses with non-isolated ventriculomegaly, and in fetuses with mild isolated ventriculomegaly.

**Results:**

16 studies were included with a total of 2137 cases of affected fetuses that met the inclusion criteria for analysis. Combined data from these studies revealed an overall incremental yield of CMA over karyotyping of 7% (95% CI 4–10%) in cases with ventriculomegaly, 4% (95% CI 2–6%) in isolated cases, 10% (95% CI 6–16%) in non-isolated cases, and 2% (95% CI 1–4%) in mild isolated cases.

**Conclusions:**

Our findings may be useful in clinical practice to guide management options and the counseling of the couples to individualize patient care and facilitate clinicians when they come across such a common clinical entity.

**Supplementary Information:**

The online version contains supplementary material available at 10.1007/s00404-026-08470-8.

## Introduction

Fetal ventriculomegaly (VM) is a condition characterized by the enlargement of the fetal cerebral ventricles, commonly detected during prenatal ultrasound assessment, with its estimated prevalence ranging from 0.3% to 2% of pregnancies. [1] Its diagnosis is established based on reference ranges first published by Cardoza et al., according to which the upper limit of fetal ventricular measurement remains unchanged during gestation corresponding to 10 mm. [2] The clinical importance of VM lies in the fact that its detection may indicate the presence of associated malformations of the brain or other organs, as well as underlying syndromes. Mild forms of VM are often normal variants associated with normal brain anatomy and generally remain stable during gestation. [3, 4] In certain instances, though, VM results from an obstruction in the cerebrospinal fluid channel or is the consequence of other cerebral abnormalities (developmental anomalies of the prosencephalon, neural proliferation, or neuronal migration disorders), making a thorough fetal anatomic evaluation necessary [5, 6].

The presence of fetal VM has been also associated with underlying chromosomal and genetic aberrations with their incidence varying and being highly dependent on the severity of VM (mild or severe) and on the presence of additional structural anomalies (isolated or non-isolated). Mild VM has been demonstrated to increase ninefold the likelihood of trisomy 21 over the general population [3, 7], while data regarding severe and non-isolated cases of VM are quite heterogeneous and, as such, though the underlying background risk is certainly increased, a specific and individualized risk assessment usually cannot be provided.[4, 8–10] In these cases, the use of conventional karyotyping to identify significant aneuploidies such as trisomy 21 is well established. However, this approach is unable to detect pathogenic copy number variants (CNVs), submicroscopic gains, or losses of DNA, which have been associated with fetal VM. [11] Over the recent years, chromosomal microarray analysis (CMA),which is a molecular approach that identifies copy number variants with a resolution of 10 Kb or more, has been introduced as a necessary and extended tool of the prenatal assessment [12, 13].

In view of the implementation of CMA in the prenatal assessment by several centers, especially after the strong recommendation toward its use by the American College of Obstetricians and Gynecologists when a fetal anomaly is detected [13, 52, 52 aim of our study was to conduct a systematic literature review and a meta-analysis to evaluate the incremental yield of chromosomal microarray analysis compared to conventional karyotyping in cases of fetal VM. We further aimed to assess CMA’s incremental yield in cases when VM was isolated, in cases when VM was associated with additional structural anomalies (non-isolated VM), and in cases with mild, isolated VM.

## Methods

This systematic review and meta-analysis was designed according to the Preferred Reporting Items for Systematic Reviews and Meta-Analyses (PRISMA) guidelines, as well as the MOOSE Guidelines for Meta-Analyses and Systematic Reviews of Observational Studies. This review was registered in the PROSPERO international database for systematic reviews (reference: CRD420261300651).

### Eligibility criteria

The present systematic review included all observational studies (prospective/retrospective cohort, case–control, nested case–control, and cross-sectional) that reported the results of CMA testing in fetuses diagnosed with fetal ventriculomegaly (both isolated and non-isolated), fetuses with ventriculomegaly without additional findings (isolated ventriculomegaly), fetuses with ventriculomegaly with additional structural findings (non-isolated ventriculomegaly), and fetuses with mild isolated ventriculomegaly. Case reports, small case series, letters to the editor, animal studies, and review articles were not included. Conference proceedings and abstracts were also excluded, as they lack important information that is necessary for the assessment of study limitations and quality of evidence.

### Information sources and search strategy

**Fig. 1:**
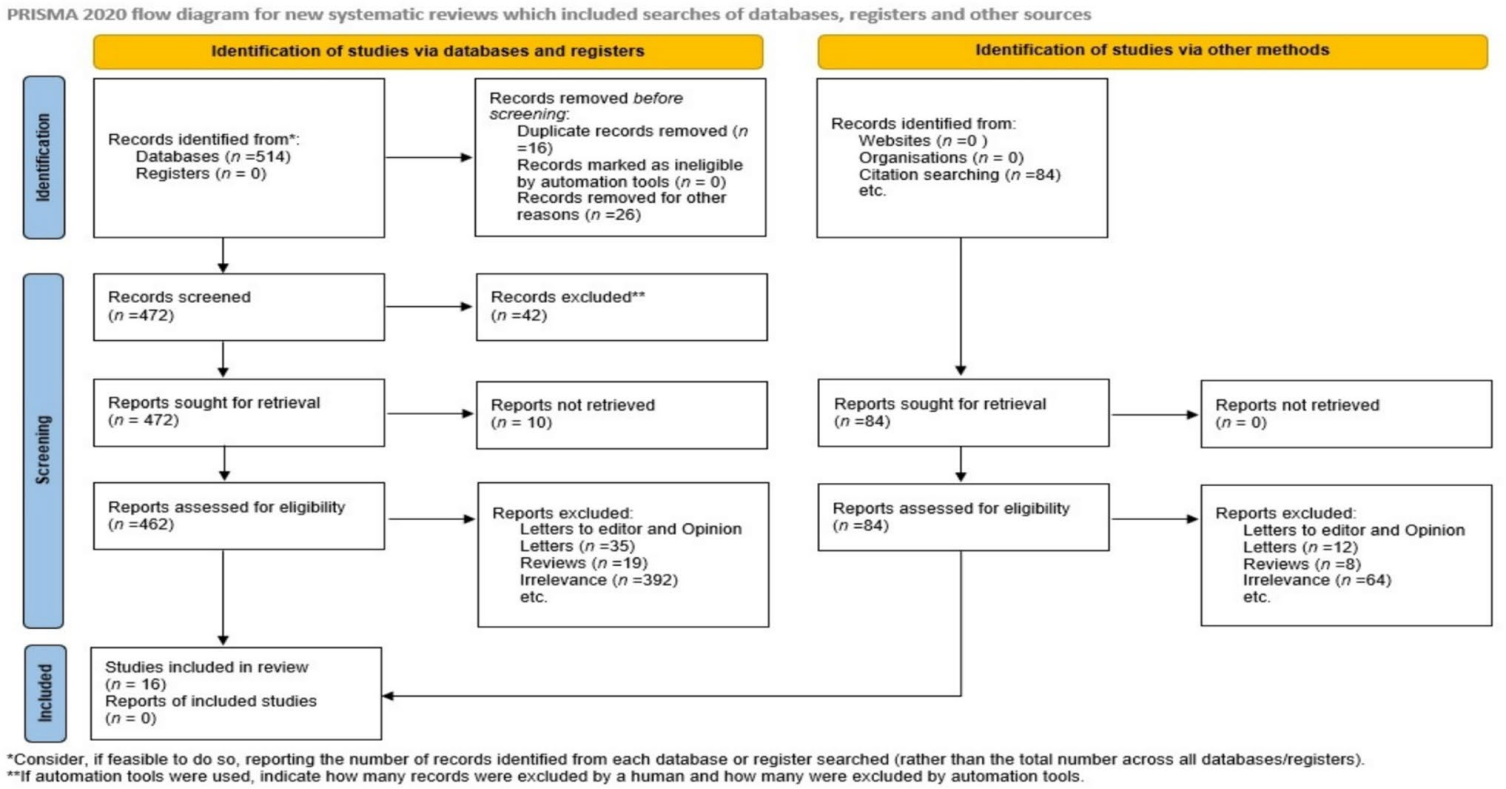
Search strategy

The Medline (1966–2025), Scopus (2004–2025), Clinicaltrials.gov (2008–2025), EMBASE (1980–2025), Cochrane Central Register of Controlled Trials CENTRAL (1999–2025), and Google Scholar (2004–2025) databases were used in our primary search, along with the reference lists of electronically retrieved full-text papers. The date of our last search was set at 30 November 2025. Our search strategy included the text words “fetal ventriculomegaly” or “ventricular dilatation” or “fetal VM” or “ultrasound anomaly” and “array comparative genomic hybridization” or “copy number variation” and is briefly presented in Fig. 1. The main search algorithm was as follows: (“fetal ventriculomegaly” OR “ventricular dilatation”OR “fetal VM” OR “ultrasound anomaly”) AND (“array comparative genomic hybridization” OR ‘aCGH’ OR “copy number variation”). The search identified 514 potentially relevant studies, but 498 were excluded because they were non-relevant articles, reviews, opinion letters, or letters to the editor, or we were unable to retrieve the available data after contacting the authors. Thus, in total, only 16 peer-reviewed papers were considered for inclusion in our systematic review and in the current meta-analysis.

### Study selection

The study selection process involved three consecutive stages. First, the titles and abstracts of all electronic papers were screened to assess their potential eligibility. Subsequently, all articles that met or were presumed to meet the eligibility criteria were retrieved as full texts. Finally, all observational (both prospective and retrospective) studies that reported the results of CMA testing in fetuses with ventriculomegaly (both isolated and non-isolated), fetuses with ventriculomegaly without additional findings (isolated cases), fetuses with additional structural findings (non-isolated cases), and fetuses with mild isolated ventriculomegaly were deemed eligible. Study selection was performed by two authors independently, while any potential discrepancies were resolved through their consensus (Fig. 1).

### Data collection

The following data were extracted from each included study: name of the first author, year of publication, study design, recruitment period, inclusion and exclusion criteria, ventriculomegaly definition, mild ventriculomegaly definition, associated anomalies that resulted in pathogenic or likely pathogenic CNVs (where applicable), and the type of CMA utilized.

When important data were missing, attempts were made to contact corresponding authors. Data extraction was performed by three authors, and any possible disagreements were resolved through consensus or by discussion with all authors. The methodological characteristics of the included studies are depicted in Table 1.

**Table 1:** Methodological characteristics of the included studies

Author, year	Type of study	Recruitment period	Inclusion criteria	Exclusion criteria	VM Definition	Mild VM Definition	Associated anomalies	CMA type
Shaffer et al. 2012 [15]	Retrospective cohort	2004–2011	Abnormal US findings	No reported results No invasive testing Abnormal karyotype at the time of CMA testing Family history of chromosomal rearrangement in one of the parents	NR	NR	NR	High-resolution BAC array
Donnelly et al. 2014 [16]	Retrospective cohort	NR	Structural anomaly NT ≥ 3.5 mm NF ≥ 6 mm Cystic hygroma	Presence of soft markers No invasive testing Missing data	≥ 10 mm	10–15 mm	CNS anomalies CVS anomalies Thoracic anomalies Skeletal anomalies Spinal anomalies Facial anomalies GIT anomalies Urinary tract anomalies Amniotic fluid anomalies Genital anomalies Nuchal anomalies Umbilical cord anomalies Pleural effusion	NR
Bardin et al. 2017 [17]	Retrospective cohort	2005–2015	Abnormal US findings beyond ≥ 23 weeks’ gestation Low risk for aneuploidies AGA fetus No prior indication for invasive testing	Major fetal malformations Sonographic soft markers prior to 23 weeks’ gestation Screen positive results for fetal aneuploidies Non-genetic indications for amniocentesis Lack of anomaly screening tests No invasive testing	≥ 10 mm	NR	Skeletal anomalies CNS anomalies GIT anomalies CVS anomalies Urinary tract anomalies Amniotic fluid anomalies FGR	CytoChip ISCA 8 × 60
Duan et al. 2019 [18]	Retrospective cohort	2013–2017	Isolated mild VM	Non-isolated VM Severe VM No invasive testing	≥ 10 mm	10–15 mm	NR	Affymetrix CytoScan 750 K
Yi et al., 2019 [19]	Retrospective cohort	2009–2017	VM Singleton pregnancies	Missing data No invasive testing Fetal infection Multiple pregnancies	≥ 10 mm	10–12 mm	NR	Affymetrix CytoScan 750 K
Chang et al. 2020 [20]	Retrospective cohort	2014–2019	VM Singleton pregnancies	No reported results Multiple pregnancies No invasive testing	≥ 10 mm	10–15 mm	CNS anomalies CVS anomalies Skeletal anomalies Increased NF Urinary tract anomalies GIT anomalies Amniotic fluid anomalies	Infinium™ OmniZhongHua-8 v1.3 BeadChips
Huang et al., 2020	Retrospective cohort	2015–2019	VM 10–15 mm Singleton pregnancies No family history	Multiple pregnancies VM > 15 mm No invasive testing Family history of congenital anomaly	≥ 10 mm	10–15 mm	CVS anomalies CNS anomalies Skeletal anomalies FGR	Oligo aCGH kit
Santirocco et al.,2020	Retrospective cohort	2009–2017	CNS anomalies Underwent CMA testing	Abnormal QF-PCR results Fetal infections No invasive testing	≥ 10 mm	NR	NTD Holoprosencephaly Posterior fossa abnormalities Cortical development anomalies Hypoxic–ischemic or hemorrhagic lesions	CytoSure Constitutional 8 × 60 K v3 qChip Pre 8 × 60 K
Wang et al., 2020 [23]	Retrospective cohort	2014–2018	Isolated and non-isolated VM	Missing data No invasive testing	≥ 10 mm	10–15 mm	CVS anomalies CNS anomalies Presence of soft markers Urinary tract anomalies	Affymetrix CytoScan 750 K
Lok et al., 2021 [24]	Retrospective cohort	2014–2018	Isolated and non-isolated VM	Missing data No invasive testing	≥ 10 mm	10–12 mm	CNS anomalies CVS anomalies Urinary tract anomalies FGR Facial anomalies Skeletal anomalies	Affymetrix CytoScan 750 K Perkin Elmer CGX V2.0
Hu et al., 2021 [25]	Prospective cohort	2014–2019	Presence of isolated soft markers	NT ≥ 3.0 mm Positive NIPT result Structural anomalies No invasive testing	≥ 10 mm	NR	N/A	Affymetrix CytoScan 750 K
Yue et al., 2024 [26]	Retrospective cohort	2018–2022	Isolated and non-isolated mild to moderate VM	Severe VM No invasive testing	≥ 10 mm	10–12 mm	GIT anomalies CVS anomalies Skeletal anomalies Presence of soft markers Polyhydramnios	Affymetrix CytoScan 750 K
Chen et al., 2024 [27]	Retrospective cohort	2022–2023	Abnormal US findings No contraindications for invasive testing Possibility of maternal cell contamination ruled out	No invasive testing Missing data	≥ 10 mm	10–15 mm	CVS anomalies Presence of soft markers Urinary tract anomalies Abdominal wall anomalies	Affymetrix CytoScan 750 K
Liu et al., 2025 [29]	Retrospective cohort	2022 – 2024	Presence of soft markers	Missing data No invasive testing	≥ 10 mm	10–15 mm	N/A	Affymetrix CytoScan 750 K
Zeng et al., 2025 [29]	Retrospective cohort	2019–2024	Isolated and non-isolated mild to moderate VM	Missing data No invasive testing	≥ 10 mm	10–12 mm	Soft markers Pleural effusion Urinary tract anomalies GIT anomalies CNS anomalies CVS anomalies	Affymetrix CytoScan 750 K
Bütün et al., 2025 [30]	Retrospective cohort	2020–2023	Structural anomalies or soft markers Singleton pregnancies	Abnormal QF-PCR results Multiple pregnancies Placental mosaicism No reported results Missing data No invasive testing	NR	NR	NR	Affymetrix Cytoscan Optima Suite method

*NR* not reported, *NA* not applicable, *FGR* fetal growth restriction, *CMA* chromosomal microarray analysis, *US* ultrasound, *NT* nuchal translucency, *NF* nuchal fold, *VM* ventriculomegaly, *CVS* cardiovascular system, *CNS* central nervous system, *GIT* gastrointestinal tract, *AGA* appropriate growth for the gestational age, *QF*-*PCR* quantitative fluorescence polymerase chain reaction, *CGH* comparative genomic hybridization, *NIPT* non-invasive prenatal testing

### Quality assessment

The methodological quality of all the included studies was evaluated using the Newcastle–Ottawa scale (NOS) tool, which is a widely utilized instrument for evaluating the methodological quality of non-randomized studies that are incorporated in systematic reviews and/or meta-analyses. The tool assesses each study based on eight criteria, which are divided into three categories: the selection of study groups; the comparability of the groups, which was based on the maternal weight and the gestational age; and the determination of either the exposure or outcome of interest for case–control or cohort studies, respectively. Stars were assigned to each quality item as a means of providing a rapid visual evaluation. Stars were allocated in a manner that granted the highest caliber studies a maximum of nine stars. The tool was implemented by two authors independently, and any discrepancies were resolved through discussion with a third author. Overall, the risk of bias was assessed to be good (7–9 stars), fair (4–6 stars), or poor (0–3 stars) [14] (Table 2).

**Table 2:** Quality assessment summary of the included studies using the Newcastle–Ottawa Assessment Scale (NOS) checklist

Newcastle–Ottawa Assessment Scale									
Author, date	Selection				Comparability	Outcome			Total
	Representativeness of the exposed cohort	Selection of the non- exposed cohort	Ascertainment of exposure	Outcome of interest not present at start of study		Assessment of outcome	Adequacy of duration of follow-up	Adequacy of completeness of follow-up	
Shaffer et al., 2012 [15]	√	√	√	√	√	√	√	–	7
Donnelly et al.,2014 [16]	√	√	√	√	√	√	–	–	6
Bardin et al., 2017 [17]	√	√	√	√	√	√	–	–	6
Duan et al., 2019 [18]	√	√	√	√	√	√	–	–	6
Yi et al., 2019 [19]	√	√	√	√	√	√	√	√	8
Chang et al., 2020 [20]	√	√	√	√	√	√	–	–	6
Huang et al., 2020	√	√	√	√	√	√	–	–	6
Santirocco et al., 2020 [22]	√	√	√	√	√	√	√	–	7
Wang et al., 2020 [23]	√	√	√	√	√	√	√	–	7
Lok et al., 2021 [24]	√	√	√	√	√	√	√	√	8
Hu et al., 2021 [25]	√	√	√	√	√√	√	√	–	8
Yue et al., 2024 [26]	√	√	√	√	√	√	√	√	8
Chen et al., 2024 [27]	√	√	√	√	√	√	–	–	6
Liu et al., 2025 [29]	√	√	√	√	√	√	–	–	6
Zeng et al., 2025 [29]	√	√	√	√	√	√	√	–	7
Butun et al., 2025	√	√	√	√	√	√	–	–	6

### Data synthesis

The incremental yield of CMA was defined as the proportion of fetuses with additional clinically significant findings (pathogenic and/or likely pathogenic) detected by CMA (CNV < 10 Mb at microarray analysis) beyond conventional karyotyping. Variants of uncertain significance were excluded from this study. Given that this represents a single proportion rather than paired diagnostic accuracy measures, a univariate random-effects meta-analysis of proportions was performed through RStudio (RStudio, Inc., Boston, MA). Proportions were stabilized using a logit transformation, and pooled estimates were calculated using inverse-variance weighting. Between-study heterogeneity was estimated using the DerSimonian–Laird method. A random-effects model was selected a priori to account for anticipated clinical and methodological heterogeneity across studies. Prediction intervals were calculated to assess the expected range of effects in future settings. Publication bias was assessed using funnel plot inspection and Egger’s regression test on logit-transformed proportions. (Supplementary Information, Figure S1).

Potential sources of heterogeneity were investigated using random-effects meta-regression models based on logit-transformed proportions. Potential moderators included isolation status, publication year, sample size, and geographic region. Between-study variance was estimated using restricted maximum likelihood. (Supplementary Information, Figures S2–S5).

### Subgroup analysis

We planned to conduct a subgroup analysis based solely on high-quality studies after performing the appropriate quality assessment (NOS score of 7–9 stars) and after the exclusion of studies that involved women with an increased a priori risk for genetic aberrations prior to the detection of fetal ventriculomegaly (women with increased first-trimester nuchal translucency measurements) [16].

## Results

Our search identified 514 potentially relevant studies, but 498 were excluded after reviewing the titles and the abstracts and after the exclusion of non-relevant articles, case reports, opinion letters, reviews, and letters to the editor. Overall, 16 studies were included in the present systematic review (16 retrospective cohorts) that enrolled a total of 2137 patients. [15–30] The search strategy is briefly presented in Fig. 1.

The methodological characteristics of the included studies are presented in Table 1 and include the inclusion and exclusion criteria, the VM definition, the associated structural anomalies that define VM cases as non-isolated, and the type of microarray used in each study. Among the 16 included studies, 12 provided specific data for the assessment of the incremental yield of CMA in cases of isolated VM [16–19, 21, 23–29], 7 provided data specifically in cases of non-isolated VM [16, 20–24, 27], and 6 provided data specifically for mild isolated cases of VM [19, 23–27]. VM definition was provided in 14 studies [17–30] and that of mild VM was provided in 11 studies [17, 19–22, 24, 25, 27–30].

**Fig. 2:**
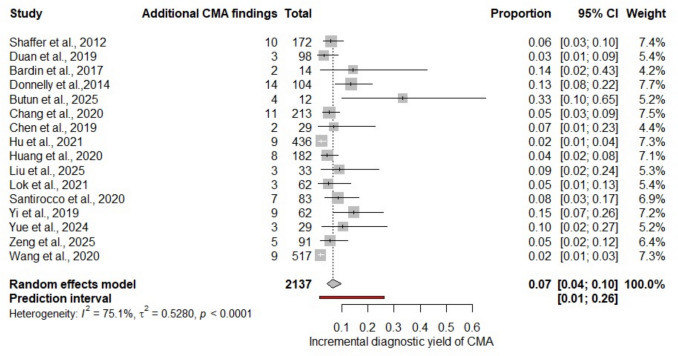
Forest plot of incremental yield by chromosomal microarray analysis (CMA) over karyotyping in fetuses with ventriculomegaly with 95% confidence intervals (CIs) and weighted pooled summary statistics using the DerSimonian–Laird method. Forest plot analysis: vertical dashed line = pooled proportion from the random-effects model. Squares = study-specific proportions with size proportional to study weight. Diamond = pooled incremental yield of CMA over karyotyping and 95% CI for all studies; Horizontal black lines = 95% CI. Horizontal red line = prediction intervals

**Fig. 3:**
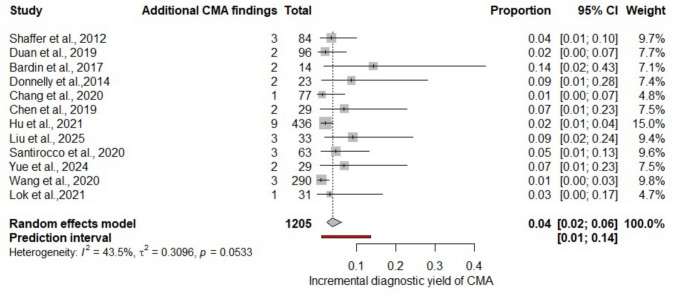
Forest plot of incremental yield by chromosomal microarray analysis (CMA) over karyotyping in cases with isolated ventriculomegaly with 95% confidence intervals (CIs) and weighted pooled summary statistics using the DerSimonian–Laird method. Forest plot analysis: vertical dashed line = pooled proportion from the random-effects model. Squares = study-specific proportions with size proportional to study weight. Diamond = pooled incremental yield of CMA over karyotyping and 95% CI for all studies. Horizontal black lines = 95% CI. Horizontal red line = prediction intervals

**Fig. 4:**
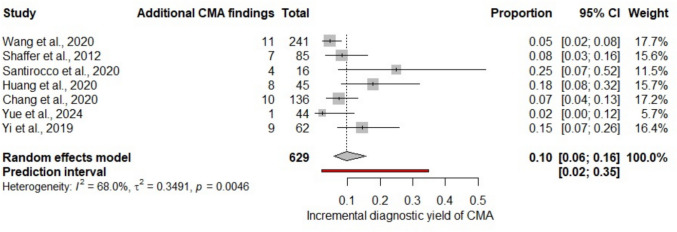
Forest plot of incremental yield by chromosomal microarray analysis (CMA) over karyotyping in non-isolated cases of ventriculomegaly with 95% confidence intervals (CIs) and weighted pooled summary statistics using the DerSimonian–Laird method. Forest plot analysis: vertical dashed line = pooled proportion from the random-effects model. Squares = study-specific proportions with size proportional to study weight. Diamond = pooled incremental yield of CMA over karyotyping and 95% CI for all studies. Horizontal black lines = 95% CI. Horizontal red line = prediction interval

**Fig. 5:**
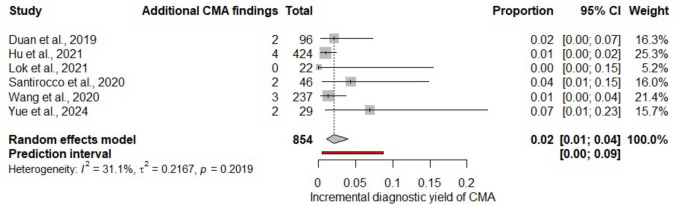
Forest plot of incremental yield by chromosomal microarray analysis (CMA) over karyotyping in mild isolated cases of ventriculomegaly with 95% confidence intervals (CIs) and weighted pooled summary statistics using the DerSimonian–Laird method. Forest plot analysis: Vertical dashed line = pooled proportion from the random-effects model. Squares = study-specific proportions with size proportional to study weight. Diamond = pooled incremental yield of CMA over karyotyping and 95% CI for all studies. Horizontal black lines = 95% CI. Horizontal red line = prediction intervals

The forest plots of the 16 included studies and the pooled results from the meta-analysis in cases of ventriculomegaly, isolated ventriculomegaly, non-isolated ventriculomegaly, and mild isolated ventriculomegaly are depicted in Figs. 2, 3, 4, and 5 respectively.

The pooled data from the reviewed studies show an overall 7% incremental yield of CMA over karyotyping (95% CI 4–10%, I^2^ = 75.1%) in cases with ventriculomegaly **(**Fig. 2**)**. The prediction interval (1–26%) suggests considerable variability in the incremental yield of CMA across studies.

The pooled data demonstrated an overall 4% incremental yield (95% CI 2–6%, I^2^ = 43.5%) in isolated cases **(**Fig. 3**)** The prediction interval (1–14%) indicated that the incremental yield of CMA was low, but varied across settings.

The pooled data demonstrated an overall 10% incremental yield (95% CI 6–16%, I^2^ = 68%) in non-isolated cases **(**Fig. 4**)**. The prediction interval (2–35%) indicated considerable variability in the incremental yield of CMA across studies.

The pooled data demonstrated an overall 2% incremental yield in mild isolated cases (95% CI 1–4%, I^2^ = 31.1%) **(**Fig. 5**)**. The prediction interval (0–9%) indicated consistently low incremental yield of CMA in mild isolated ventriculomegaly.

The observed incremental yield for each single study ranged from 2 to 33% in cases of VM, from 1 to 14% in isolated cases, from 2 to 25% in non-isolated cases, and from 0 to 7% in mild isolated cases.

The fetal anomalies along with the clinically significant submicroscopic CNVs they were associated with in non-isolated cases of fetal VM are presented in Table 3.

**Table 3:** Pathogenic CNVs and fetal anomalies identified by CMA analysis in fetuses with non-isolated ventriculomegaly and normal karyotype

(Author, -year)	Total	Events	Ultrasound findings	CNVs
Shaffer (2012) [15]	85	7	NR	NR
Yi (2019) [19]	62	9	NR	NR
Huang (2020)	45	8	Widening of the third ventricle	15q11.1q11.2(20,481,702–23,672,681) × 3
			Lissencephaly	17p13.3-p13.2(87,009–3,418,004) × 1 17p13.3-(1,537,730–2,537,850) × 1
			Cortical malformation	5q35.2- q35.3(175,576,602–177422760) × 1 9p13.1-p11.2(39,254,329–43,704,969) × 1
			Partial absence of the corpus callosum Dandy–Walker malformation Tethered cord syndrome	6q25.2- q25.3( 154,827,149–158,734,846) × 1
			Cortical malformation, absence of corpus callosum lissencephaly	1q43-1q44(2,402,100,011–249,210,000) × 1
			Partial absence of corpus callosum	17p13.3-p13.1(87,009–6,823,208) × 1
Chang (2020) [20]	136	10	Posterior fossa widening Dilation of the third ventricle	arr[hg19]18p11p11.23(7,024,998–7,578,782) × 3
			Posterior fossa cyst	arr[hg19]2p24.3(13,579,747 e14,334,195) × 3
			Posterior fossa cyst; bilateral renal pelvis separation	arr[hg19]2p25.2p25.3(4,124,656 e4,641,479) × 1
			Ependymal cyst; echogenic bowel; bowel dilation	arr[hg19] 8p23.2(2,355,745—2,801,788) × 3
			Ascending aorta dilatation; S/D ratio increased	arr[hg19]15q22.31q23(66,981,329 e68,050,777) × 1
			Posterior fossa cistern cyst; dilation of the fourth ventricle; Cavum vergae cyst Cerebellum dysplasia; cardiomegaly; tricuspid regurgitation; pulmonary valve Stenosis with regurgitation; foramen ovale dilation; fetal growth restriction; echogenic bowel	arr[hg19]11p12(40,966,294 e43,095,376) × 1 arr[hg19] Xp22.2p22.13(16,950,577 e17,711,019) × 3
			Choroid plexus cyst; cavum septum pellucidum cyst	arr[hg19]16p13.11p13.12(14,760,734 e16,303,388) × 1
			Posterior fossa widening; polyhydramnios	arr[hg19]2q12.2q12.3(106,878,354 e108,440,138) × 3; arr[hg19]6q21(111,673,714e112,732,041) × 3
Santirocco (2020) [22]	16	4	Congenital diaphragmatic hernia, renal cyst	Xq26.2(132,315,039 _132876911) × 0
			Hydrops; polyhydramnios	17q12(34856055_ 36,777,884) × 1
			Tetralogy of Fallot; microretrognathia; clubfoot	2q21.2(134067405_ 134,295,740) × 1 14q11.2(22409129_ 23,167,893) × 3
Yue (2024) [26]	44	1	Persistent left superior vena cava; polydactyly	10q11.22q11.23 (45,788,343- 50,241,236) × 3
Wang (2020) [23]	241	11	Short femur length	arr 16p11.2 (29581101_30190029) × 3
			Polyhydramnios	arr 16p13.12p12.3 (14512852_17637607) × 3
			Echogenic kidney	arr 17q12 (34822465_36307773) × 1
			Cerebral dysplasia	arr 22q11.21 (18919477_21454872) × 3
			Absent gallbladder, intracardiac echogenic focus	arr 12p13.33 (173786_1606351) × 1
			Talipes equinus	arr 12q24.21 (116025185_116416736) × 1
			Hypoplasia of the corpus callosum	arr 3q28q29 (190376787_197851444) × 3 arr 17p13.3 (525_2780094) × 1
			Hypoplasia of the corpus callosum	arr 22q11.21 (18919477_21800471) × 3
			Hydronephrosis	arr 5p15.33p15.31 (113576_9308549) × 3
			Cerebellar hypoplasia, enlarged cisterna magna, Cystic hygroma, cleft lip	arr 13q33.2q34(106367669_115107733) × 4

*NR* not reported

### Meta-regression analysis results

Meta-regression analyses were performed to explore potential sources of between-study heterogeneity investigating the isolation status, the publication year, the geographic region and the study sample size. (Supplementary Information, Figures S2–S5).

Isolation status was not a significant moderator of incremental diagnostic yield (*β* =  − 0.46, 95% CI: −  1.46 to 0.55; p = 0.37) and explained only a negligible proportion of heterogeneity (R^2^ = 1.4%). (Supplementary Information, Figure S2).

No significant association was observed in terms of publication year (*β* = 0.01, 95% CI  − 0.11 to 0.13; *p* = 0.87) and no temporal trend was identified (Supplementary Information, Figure S3).

Geographic region was not associated with incremental yield (QM = 0.53; *p* = 0.77) with no significant differences between studies conducted in Asia, Europe, or the USA and no reduction in between-study heterogeneity (R^2^ = 0%). (Supplementary Information, Figure S4).

Study sample size was a significant moderator (*β* =  − 0.68, 95% CI: − 0.94 to − 0.43; *p* < 0.001) with smaller studies reporting higher incremental diagnostic yields. Inclusion of sample size in the model explained a substantial proportion of heterogeneity (*R*^2^ = 85.7%) and residual heterogeneity was no longer statistically significant (*I*^2^ = 29.4%, *p* = 0.18). (Supplementary Information, Figure S5).

### Subgroup analysis results

**Fig. 6:**
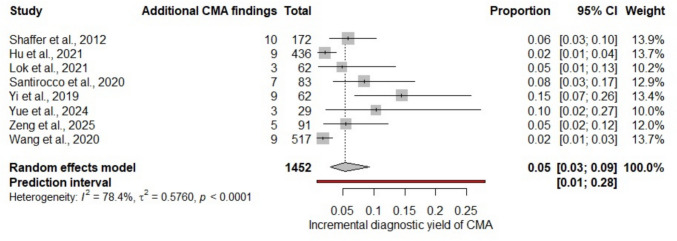
Subgroup analysis—forest plot of incremental yield by chromosomal microarray analysis (CMA) over karyotyping in cases of ventriculomegaly with 95% confidence intervals (CIs) and weighted pooled summary statistics using the DerSimonian–Laird method. Forest plot analysis: vertical dashed line = pooled proportion from the random-effects model. Squares = study-specific proportions with size proportional to study weight. Diamond = pooled incremental yield of CMA over karyotyping and 95% CI for all studies. Horizontal black lines = 95% CI. Horizontal red line = prediction intervals

The subgroup analysis, which was limited to eight high-quality studies, included a total of 1452 fetuses with ventriculomegaly. The pooled data demonstrated an overall 5% incremental yield (95% CI 3–9%, I^2^ = 78.4%) in cases with ventriculomegaly **(**Fig. 6**)**. The prediction interval (1–28%) indicates considerable variability in the incremental yield of CMA across studies.** (**Fig. 6**).**

## Discussion

### Principal findings of our study

The study demonstrated: first, an overall 7% incremental yield of CMA over karyotyping (95% CI 4–10%, I^2^ = 75.1%) in cases with ventriculomegaly; second, an overall 4% incremental yield (95% CI 2–6%, *I*^2^ = 43.5%) in isolated cases; third, an overall 10% incremental yield (95% CI 6–16%, *I*^2^ = 68%) in non-isolated cases; and fourth, an overall 2% incremental yield in mild isolated cases (95% CI 1–4%, *I*^2^ = 31.1%). In all cases, the prediction intervals indicated considerable variability across studies.

### Comparison to existing literature

The incremental yield of CMA over standard karyotyping in fetuses with VM in our study (7%, 95% CI 4–10%) differs from the one previously published by Sun et al. [31] (11%, 95% CI 7%–16%), a fact that may be explained by the inclusion of both pathogenic submicroscopic chromosomal abnormalities and variants of unknown clinical significance in their case. Moreover, this previous meta-analysis is based on 8 studies and a total sample of 946 affected fetuses with their data mostly originating from China, which, according to the authors, demonstrates a high incidence of congenital anomalies and birth defects, a finding that may have an impact on the generalizability of their findings [32].

The clinical significance of CMA as part of routine prenatal assessment was reported by Wapner et al., with the authors demonstrating that fetuses with normal karyotype and without structural anomalies may carry clinically significant copy number variations in 1.7% of cases. [33] Similar results were demonstrated later by a meta-analysis by Srebniak et al., which included 10 studies and a total of 10,641 structurally normal fetuses, with a submicroscopic pathogenic abnormality identified prenatally in 0.84% of the included fetuses. [34] The additive value of CMA has also been extensively investigated in fetuses exhibiting structural anomalies, with literature indicating that CMA will yield supplementary information beyond karyotype in around 6%–7% of pregnancies with fetal anomalies [35–37]. However, it should be underlined that the incremental yield of CMA varies significantly upon the specific organ system affected and the presence of either single or multiple defects. In this view, the anomalies most frequently linked to abnormal CMA results are the cardiac, skeletal, urogenital, renal, and central neurological systems [38–41].

In terms of central nervous system findings, the significance of ventriculomegaly ranges from being a totally benign normal variant to being the endpoint of several underlying pathological cerebral processes or the consequence of an underlying spinal dysraphism. Even though both the American College of Obstetricians and Gynecologists (ACOG) and the Society for Maternal–Fetal Medicine (SMFM) suggest diagnostic testing with CMA in case of VM detection, the data already published seem to be quite heterogeneous and are probably influenced by the severity of VM and the anomalies that VM is accompanied by. [42, 43] To be more precise, the recent study by Bütün et al., which included 12 cases of ventriculomegaly with normal conventional karyotyping, revealed four cases of pathogenic submicroscopic CNVs which represent a 33% incremental yield. However, the authors do not make it clear whether this included fetuses with isolated ventriculomegaly only or if those with additional structural abnormalities or soft markers were included as well. [30] Similarly, Yi et al. demonstrated an incremental yield of 14% without clearly differentiating isolated from non-isolated cases of VM. [19] On the other hand, when studies investigated the incremental yield of CMA in isolated cases of VM, its additive value appeared much lower. A recent study by Wang et al., which included a cohort of 548 fetuses diagnosed with VM, showed that the number of chromosomal aberrations was much lower in isolated cases compared to the non-isolated ones (2.38% vs 12.99%, *p* < 00001) yielding an incremental yield of 1%. [23] Similar findings were demonstrated by Lok et al. In their cohort of 46 fetuses with isolated VM, only 1 (1/46, 2.1%) had a submicroscopic pathogenic aberration. Furthermore, in their study, the prevalence of abnormal karyotype or CMA results was not significantly affected by the degree of ventricular dilatation [24]. This finding implies that the presence or absence of associated anomalies has a greater effect on the prevalence of underlying genetic aberrations than does the severity of VM. This is in line with our results which showed that the incremental yield of CMA over karyotype rises from 2% in isolated cases to 8% in non-isolated ones.

Among the included studies, the additive value of CMA is even smaller in cases with mild isolated VM ranging from 0 [24] to 7% [26], yielding an overall 1%. However, this incremental yield should be interpreted with caution especially after the recent results of Srebniak et al. [35] which revealed a 0.84% risk (95% CI 0.55–1.30%) of otherwise low risk fetuses to carry a pathogenic clinically significant submicroscopic aberration.

Nevertheless, even after the extensive use of conventional karyotype and CMA, a diagnosis remains elusive in numerous instances. As such, supplementary diagnostic modalities might be essential to ascertain the underlying genetic etiology of VM and to offer the appropriate counseling and guidance. In this view, prenatal exome sequencing (p-ES) has emerged and has been assessed by several studies in cases in which both conventional karyotype and CMA provide normal results. [44–46] It is necessary to underscore that the review and meta-analysis conducted by Blayney et al. demonstrated an additional 20% incremental yield of p-ES analysis in cases of mild VM, an additional 22% incremental yield in cases with moderate VM and an additional 20% in cases with severe VM. [47] Those findings are potentially making p-ES analysis a valuable diagnostic asset not only for the investigation of severe congenital anomalies [48] but also for VM. However, this approach should be further investigated and clinically assessed by larger studies, which take into account both the severity of VM and the presence or absence of other structural anomalies.

### Strengths and limitations

To our knowledge, this is the first meta-analysis to investigate the incremental yield of CMA over karyotyping in fetuses with VM, solely focusing on pathogenic submicroscopic genetic aberrations (after the exclusion of benign, likely benign and variants of unknown significance). Additionally, we assessed and included only true submicroscopic CNVs, having excluded CNVs ≥ 10 Mb that are detectable in conventional karyotyping. Furthermore, we performed multiple subgroup analyses to assess the incremental yield of CMA in cases with isolated VM, non-isolated VM and mild isolated VM.

However, there are certain limitations to be noted. There is heterogeneity in the definition of mild VM, given the fact that four studies defined it as lateral ventricular diameter measuring 10–12 mm while in seven of the included studies VM was defined as measurement between 10 and 15 mm. As such, our mild isolated subgroup analysis might include cases that, according to other studies, fall within the moderate ventriculomegaly subgroup. Additionally, due to the unavailability of several demographic data for each individual, many influencing factors, including maternal age and gestational age at diagnosis, were not comprehensively evaluated. It should be further underlined that the heterogeneity demonstrated in cases of ventriculomegaly and in cases of non-isolated ventriculomegaly was reported to be 75.1% and 68%, respectively, which indicated considerable variability in incremental diagnostic yield across studies. This was explained by our meta-regression analysis according to which study sample size emerged as the primary determinant of heterogeneity with smaller studies consistently reporting higher incremental diagnostic yields. Such a pattern suggests the presence of small-study effects which may reflect publication bias, selective reporting or differences in study populations. (Supplementary Information, Figure S5) Furthermore, it should be noted that 11 out of the 16 included studies originate from China and, even though the geographic region was not identified as a significant source of heterogeneity, the generalizability of our findings in other ethnic groups remains uncertain (Supplementary Information, Figure S4). In view of the elevated detection rate of submicroscopic CNVs in cases with non-isolated VM, studies have indicated that several fetal abnormalities are linked to underlying genetic anomalies [49–51]. Consequently, in these cases, the added value of VM on submicroscopic aberrations remains ambiguous, considering the already elevated background risk attributed to complex structural abnormalities which may potentially also explain the heterogeneity demonstrated in our findings. As such, our results in those cases should be interpreted with caution, as it is not clear if the observed incremental yield should be attributed to VM itself or the associated structural anomaly.

## Conclusion

Our study demonstrated:, first, an overall 7% incremental yield of CMA over karyotyping in cases with ventriculomegaly; second, an overall 4% incremental yield in isolated cases; third, an overall 10% incremental yield in non-isolated cases; and fourth, an overall 2% incremental yield in mild isolated cases. Regardless of the heterogeneity demonstrated, which mostly may be explained by the presence of small-study effects, the results of our study may prove to be useful in clinical practice to guide management options and counseling of patients whose fetuses are diagnosed with the relatively common finding of ventriculomegaly.

## Supplementary Information

Below is the link to the electronic supplementary material.

Supplementary file1 (DOCX 647 KB)

## Data Availability

Data sharing is not applicable to this article as no datasets were generated or analyzed during the current study.
