# Supplementary material for: The incremental yield of CMA over karyotype in fetal ventriculomegaly: a systematic review and meta-analysis
Source: Arch Gynecol Obstet. 2026 Jun 2;313(1):243. doi: 10.1007/s00404-026-08470-8 (PMC13442320; doi:10.1007/s00404-026-08470-8)
Supplement: Supplementary file 1 [file 404_2026_8470_MOESM1_ESM.docx]

**Figure S1.** Funnel plot of the incremental diagnostic yield of chromosomal microarray analysis (CMA). Individual studies are plotted by logit-transformed proportions against their standard errors. Vertical dashed line: pooled estimate, Diagonal lines: pseudo 95% confidence limits. Visual inspection and Egger’s regression test were used to assess small-study effects.

**Linear regression test of funnel plot asymmetry**

Test result: t = 0.84, df = 14, p-value = 0.4159

Bias estimate: 1.4380 (SE = 1.7153)

Details:

- multiplicative residual heterogeneity variance (tau^2 = 4.0997)

- predictor: standard error

- weight: inverse variance

- reference: Egger et al. (1997), BMJ

**Figure S2.** Meta-regression analysis on isolated vs mixed/non-isolated studies. Meta-regression analysis did not identify isolation status as a significant source of heterogeneity (β = −0.46, 95% CI: −1.46 to 0.55; p = 0.37). Residual heterogeneity remained substantial (I² = 73.9%) and isolation status explained only a small proportion of between-study variance (R² = 1.4%).

**Meta-regression output data on isolated vs mixed/non-isolated studies.**

Mixed-Effects Model (k = 16; tau^2 estimator: REML)

logLik deviance	AIC	BIC	AICc

-17.7071	35.4143	41.4143	43.3314	43.8143

tau^2 (estimated amount of residual heterogeneity): 0.4994 (SE = 0.2690)

tau (square root of estimated tau^2 value): 0.7067

I^2 (residual heterogeneity / unaccounted variability): 73.91%

H^2 (unaccounted variability / sampling variability): 3.83

R^2 (amount of heterogeneity accounted for): 1.38%

Test for Residual Heterogeneity:

QE(df = 14) = 53.7807, p-val < .0001

Test of Moderators (coefficient 2):

QM(df = 1) = 0.7903, p-val = 0.3740

Model Results:

estimate	se	zval	pval	ci.lb	ci.ub

intrcpt	-2.5490 0.2410 -10.5787 <.0001 -3.0213 -2.0768 ***

isolatedIsolated	-0.4553 0.5122	-0.8890 0.3740 -1.4591	0.5485

---

Signif. codes: 0 ‘***’ 0.001 ‘**’ 0.01 ‘*’ 0.05 ‘.’ 0.1 ‘ ’ 1

**Figure S3.** Meta-regression analysis based on the publication year of the included studies. Publication year did not explain between-study heterogeneity (R² = 0%) and substantial residual heterogeneity remained (I² = 75.7%).

**Meta-regression output data based on the publication year of the included studies.**

Mixed-Effects Model (k = 16; tau^2 estimator: REML)

logLik deviance	AIC	BIC	AICc

-18.1245	36.2490	42.2490	44.1662	44.6490

tau^2 (estimated amount of residual heterogeneity): 0.5607 (SE = 0.2950)

tau (square root of estimated tau^2 value): 0.7488

I^2 (residual heterogeneity / unaccounted variability): 75.71%

H^2 (unaccounted variability / sampling variability): 4.12

R^2 (amount of heterogeneity accounted for): 0.00%

Test for Residual Heterogeneity:

QE(df = 14) = 58.8054, p-val < .0001

Test of Moderators (coefficient 2):

QM(df = 1) = 0.0255, p-val = 0.8732

Model Results:

estimate	se	zval	pval	ci.lb	ci.ub

intrcpt -22.3132 123.2626 -0.1810 0.8564 -263.9035 219.2770

year	0.0097	0.0610	0.1596 0.8732	-0.1099	0.1293

---

Signif. codes: 0 ‘***’ 0.001 ‘**’ 0.01 ‘*’ 0.05 ‘.’ 0.1 ‘ ’ 1

**Figure S4.** Meta-regression analysis based on the region of the included studies. Meta-regression analysis did not identify geographic region as a significant source of heterogeneity (QM = 0.53, p = 0.77). Compared with studies conducted in Asia, no significant differences were observed in studies from Europe (β = 0.35, 95% CI: −1.41 to 2.10; p = 0.70) or the United States (β = 0.42, 95% CI: −0.82 to 1.66; p = 0.51). Geographic region did not explain between-study heterogeneity (R² = 0%) and substantial residual heterogeneity remained (I² = 75.5%).

**Meta-regression output data based on the region of the included studies.**

Mixed-Effects Model (k = 16; tau^2 estimator: REML)

logLik deviance	AIC	BIC	AICc

-17.1650	34.3300	42.3300	44.5898	47.3300

tau^2 (estimated amount of residual heterogeneity): 0.5790 (SE = 0.3183)

tau (square root of estimated tau^2 value): 0.7609

I^2 (residual heterogeneity / unaccounted variability): 75.53%

H^2 (unaccounted variability / sampling variability): 4.09

R^2 (amount of heterogeneity accounted for): 0.00%

Test for Residual Heterogeneity:

QE(df = 13) = 52.6399, p-val < .0001

Test of Moderators (coefficients 2:3):

QM(df = 2) = 0.5309, p-val = 0.7668

Model Results:

estimate	se	zval	pval	ci.lb	ci.ub

intrcpt	-2.7310 0.2538 -10.7621 <.0001 -3.2283 -2.2336 ***

regionSpain	0.3462 0.8941	0.3872 0.6986 -1.4062	2.0985

regionUnited States	0.4162 0.6332	0.6573 0.5110 -0.8248	1.6573

---

Signif. codes: 0 ‘***’ 0.001 ‘**’ 0.01 ‘*’ 0.05 ‘.’ 0.1 ‘ ’ 1

**Figure S5.** Meta-regression analysis based on the sample size of the included studies. Meta-regression analysis demonstrated a significant association between study sample size and incremental diagnostic yield (β = −0.68, 95% CI: −0.94 to −0.43; p < 0.001) with smaller studies reporting higher yields. Inclusion of sample size in the model explained a substantial proportion of between-study heterogeneity (R² = 85.7%) and residual heterogeneity was no longer statistically significant (I² = 29.4%, p = 0.18).

**Meta-regression output data based on the sample size of the included studies.**

Mixed-Effects Model (k = 16; tau^2 estimator: REML)

logLik deviance	AIC	BIC	AICc

-10.5563	21.1127	27.1127	29.0299	29.5127

tau^2 (estimated amount of residual heterogeneity): 0.0727 (SE = 0.0922)

tau (square root of estimated tau^2 value): 0.2696

I^2 (residual heterogeneity / unaccounted variability): 29.36%

H^2 (unaccounted variability / sampling variability): 1.42

R^2 (amount of heterogeneity accounted for): 85.65%

Test for Residual Heterogeneity:

QE(df = 14) = 18.6424, p-val = 0.1791

Test of Moderators (coefficient 2):

QM(df = 1) = 27.3048, p-val < .0001

Model Results:

estimate	se	zval	pval	ci.lb	ci.ub

intrcpt	0.5231 0.6314	0.8285 0.4074 -0.7144	1.7606

logN	-0.6829 0.1307 -5.2254 <.0001 -0.9390 -0.4267 ***

---

Signif. codes: 0 ‘***’ 0.001 ‘**’ 0.01 ‘*’ 0.05 ‘.’ 0.1 ‘ ’ 1
